# Prevalence of depression and anxiety among undergraduate university students in low- and middle-income countries: a systematic review protocol

**DOI:** 10.1186/s13643-018-0723-8

**Published:** 2018-04-10

**Authors:** James January, Munyaradzi Madhombiro, Shalote Chipamaunga, Sunanda Ray, Alfred Chingono, Melanie Abas

**Affiliations:** 1 Department of Community Medicine, College of Health Sciences, University of Zimbabwe, Box A178, Avondale, Harare, Zimbabwe; 2 Department of Psychiatry, School of Clinical Medicine, College of Health Sciences, University of KwaZulu-Natal, Durban, South Africa; 3 Department of Psychiatry, College of Health Sciences, University of Zimbabwe, Harare, Zimbabwe; 4 Department of Health Professions Education, College of Health Sciences, University of Zimbabwe, Harare, Zimbabwe; 5 Institute of Psychiatry, King’s College London, London, UK

**Keywords:** Anxiety, Depression, Students, LMICs, Systematic review protocol

## Abstract

**Background:**

Depression and anxiety symptoms are reported to be common among university students in many regions of the world and impact on quality of life and academic attainment. The extent of the problem of depression and anxiety among students in low- and middle-income countries (LMICs) is largely unknown. This paper details methods for a systematic review that will be conducted to explore the prevalence, antecedents, consequences, and treatments for depression and anxiety among undergraduate university students in LMICs.

**Methods:**

Studies reporting primary data on common mental disorders among students in universities and colleges within LMICs will be included. Quality assessment of retrieved articles will be conducted using four Joanna Briggs critical appraisal checklists for prevalence, randomized control/pseudo-randomized trials, descriptive case series, and comparable cohort/case control. Meta-analysis of the prevalence of depression and anxiety will be conducted using a random effects model which will generate pooled prevalence with their respective 95% confidence intervals.

**Discussion:**

The results from this systematic review will help in informing and guiding healthcare practitioners, planners, and policymakers on the burden of common mental disorders in university students in LMICs and of appropriate and feasible interventions aimed at reducing the burden of psychological morbidity among them. The results will also point to gaps in research and help set priorities for future enquiries.

**Systematic review registration:**

PROSPERO CRD42017064148

## Background

Poor mental health among university students has been a cause of concern globally. A previous systematic review indicated that university students have higher rates of depression than the general population [1]. Prevalence of depression or anxiety among health professions’ students have also been reported to be higher than in the general population in resource-constrained settings [2–6] and resource-rich settings [7, 8]. Most of these studies have reported prevalence of depression or anxiety above 35% [1, 2, 4, 5]. The studies have tended to focus on common mental disorders among medical students and have largely ignored university students in other fields. Understanding the burden of psychological morbidity among university and college students is imperative as there is evidence showing that cognitive, behavioral, and mindfulness interventions can be effective in reducing anxiety and depressive symptoms in these groups [9, 10]. Such interventions are particularly useful in resource-limited settings such as low- and middle-income countries (LMICs) where antidepressants may not be easily available or the appropriate solution.

Factors implicated in psychological morbidity among students include academic pressure, demanding workloads [11], worry about own health [12], financial concerns [13], exposure to patients’ suffering in the case of medical students [14, 15], and student abuse and mistreatment [16].

Psychological distress among students may adversely influence their academic performance and quality of life [17] and may contribute to alcohol and substance abuse, decreased empathy, and academic dishonesty [18]. In light of the risks and consequences of psychological morbidity on students and the remarkable growth in university student numbers in Sub-Saharan Africa within the last 30 years [19], there is a need to understand the prevalence and antecedents of common mental disorders among university students. University/college-based mental health well-being programs and interventions become increasingly imperative as they contribute to prevention and minimization of psychological morbidity. Additionally, there is a need to create supportive environments for students who may be having mental health difficulties during their training. Previous systematic reviews evaluating the prevalence of depressive or anxiety symptoms among health professions’ students have been conducted on studies that were carried out in the USA and Canada [7] and other high-income settings and mainly confined to English-speaking countries [8] focusing on medical students. This review will collate evidence from LMICs with regard to the burden of depression or anxiety among university student populations.

### Purpose of the review

This systematic review will be conducted in an effort to answer the following key questions:

1. What is the documented prevalence of depression or anxiety among university students in LMICs?
2. Which sociodemographic and curricular factors are associated with depression or anxiety among university students in LMICs?
3. What are the reported short and medium term consequences of depression or anxiety among university students in LMICs?

## Methods

This study protocol is structured in accordance with the Preferred Reporting Items for Systematic reviews and Meta-Analyses (PRISMA). The PRISMA contains a 27-item checklist which is aimed at facilitating the development and reporting of robust systematic review protocols [20]. The systematic review was registered on the PROSPERO database (CRD42017064148).

### Information sources

PubMed, PsychINFO, EMBASE and African Index Medicus, BIREME, LILACS, and MEDLINE databases will be searched for studies reporting primary data on common mental disorders (depression and/or anxiety) among students in universities and colleges within LMICs. For this study, LMICs will be defined using the World Bank Country Lending Group list for the year 2017 [21].

### Search strategy

In light of the paucity of studies in LMICs, no time restrictions will be imposed on the search. Controlled vocabulary terms will be appropriately incorporated for each database. We will use the terms to search for three main concepts namely (1) undergraduate university/college students, (2) prevalence of depression or anxiety, and (3) low- and middle-income countries. A full search strategy for one database is displayed in Appendix 1. Reference lists of retrieved articles will also be examined and additional articles added if they meet the inclusion criteria.

### Eligibility criteria

Studies will be included if they report the prevalence of depression or anxiety among university/college students undertaking undergraduate degree programs. Study types will include descriptive and analytical studies such as cross-sectional and longitudinal studies, case-series analysis, and randomized control trials that include data on prevalence of depression or anxiety. We will include studies in all languages, which will be translated into English. Due to paucity of research on depression or anxiety in most LMICs, the studies will not be excluded based on how they measured depression or anxiety since it is important to understand how these conditions are being measured in different settings.

### Data extraction

Two reviewers will independently screen titles and then abstracts of included articles using a piloted data extraction sheet (Appendix 2). Examples of the type of data that will be extracted include study design, setting, study sample sizes, assessments used for diagnosing depression or anxiety, and prevalence of depression or anxiety. Where there will be doubts on whether a title is relevant, it will be included for retrieval. Reconciliation of disagreements on which article(s) to include will be resolved by discussion and consensus between the two reviewers, or mediation by a third person.

### Assessment of methodological quality

All retrieved papers eligible for selection will undergo an assessment process conducted by two independent reviewers. Standardized critical appraisal tools will be utilized in the quality assessment. In this study, four critical appraisal tools [22] will be used to assess for quality depending on the study design. These are as follows:

1. The Joanna Briggs Institute (JBI) Prevalence Critical Appraisal tool [23]
2. The JBI critical appraisal checklist for randomized control/pseudo-randomized trials
3. The JBI critical appraisal checklist for descriptive/case series
4. The JBI critical appraisal checklist for comparable cohort/case control.

These tools were developed primarily for use in systematic reviews. Where there are disagreements between the two reviewers, a third reviewer will be engaged and discussions among the three reviewers will be used to resolve the differences.

### Data analysis and synthesis

Meta-analysis of the prevalence of depression and anxiety among university students will be conducted using a random effects model which will generate pooled prevalence with their respective 95% CIs. Analyses will be conducted in Stata 14. The results from the review will be summarized and presented in text, Appendix 3, and tables.

## Discussion

This systematic review will be conducted as the initial step of a longitudinal study on common mental disorders among university students in Zimbabwe. The review aims to explore the prevalence, antecedents, and consequences of depression and anxiety among university students in LMICs. The results from the review will inform and guide health care practitioners and researchers on appropriate and feasible interventions aimed at enhancing the psychological well-being of undergraduate students in resource-constrained settings.

## Figures and Tables

**Fig. 1:**
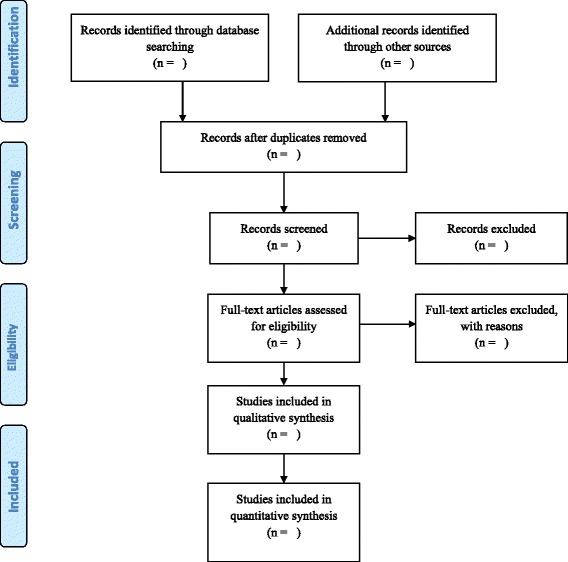
PRISMA flow diagram

## Appendix 1

### Search strategy example

MESH terms will be used to search for studies and these are “college students”, OR “university students”, OR “undergraduate students”, AND “depression” OR “mental distress” OR “common mental disorder” OR “mental disorder” OR “mental health” OR “mental illness” OR “anxiety” OR “anxiety disorder” OR “anxiety symptoms” AND Afghanistan OR Benin OR “Burkina Faso” OR Burundi OR “Central African Republic” OR Chad OR Comoros OR Congo OR Eritrea OR Ethiopia OR Gambia OR Guinea OR “Guinea-Bissau” OR Haiti OR “North Korea” OR “Democratic People’s Republic of Korea” OR Liberia OR Madagascar OR Malawi OR Mali OR Mozambique OR Nepal OR Niger OR Rwanda OR Senegal OR “Sierra Leone” OR Somalia OR “South Sudan” OR Tanzania OR Togo OR Uganda OR Zimbabwe OR Armenia OR Bangladesh OR Bhutan OR Bolivia OR “Cabo Verde” OR “Cape Verde” OR Cambodia OR Cameroon OR Congo OR “Cote D’Ivoire” OR Djibouti OR Egypt OR “el Salvador” OR Ghana OR Guatemala OR Honduras OR India OR Indonesia OR Kenya OR Kiribati OR Kosovo OR Kyrgyz OR Kyrgyzstan OR Lao OR Laos OR Lesotho OR Mauritania OR Micronesia OR Moldova OR Mongolia OR Morocco OR Myanmar OR Nicaragua OR Nigeria OR Pakistan OR “Papua New Guinea” OR Philippines OR Samoa “Sao Tome” OR Principe OR “Solomon Islands” OR “Sri Lanka” OR Sudan OR Swaziland OR Syria OR “Syrian Arab Republic” OR Tajikistan OR Timor OR Tonga OR Tunisia OR Ukraine OR Uzbekistan OR Vanuatu OR Vietnam OR “West Bank” OR Gaza OR Yemen OR Zambia OR Albania OR Algeria OR “American Samoa” OR Angola OR Argentina OR Azerbaijan OR Belarus OR Belize OR Bosnia OR Herzegovina OR Botswana OR Brazil OR Bulgaria OR China OR Colombia OR “Costa Rica” OR Cuba OR Dominica OR “Dominican Republic” OR “Equatorial Guinea” OR Ecuador OR Fiji OR Gabon OR Georgia OR Grenada OR Guyana OR Iran OR Iraq OR Jamaica OR Jordan OR Kazakhstan OR Lebanon OR Libya OR Macedonia OR Malaysia OR Maldives OR “Marshall Islands” OR Mauritius OR Mexico OR Montenegro OR Namibia OR Palau OR Panama OR Paraguay OR Peru OR Romania OR Russia OR Russian OR Serbia OR “South Africa” OR “St. Lucia.”

## Appendix 2

**Table 1:** Data extraction codebook

Data item	Operational definition	Example
Study design	Cross-sectional studies, longitudinal studies, case-series analysis, randomized control trials that include baseline data on prevalence of depression or anxiety	
Setting/source of sample/country	Universities/colleges offering health-related courses	
	Primary care settings, hospital	
	Community	
	Student health centers	
	LMICs	
Outcomes	Prevalence of depression or anxiety or mixed	
Assessment methods	Clinical interview (specify practitioner) vs.	
	Tools (specify tool)	
Sample size	Males	
	Females	
Sampling method	Any	
Response rate		
Field of training		

## Abbreviations

BIREME: Biblioteca Regional de Medicina
EMBASE: Excerpta Medica dataBASE
JBI: Joanna Briggs Institute
LILACS: Latin American and Caribbean Health Sciences Literature
LMICs: Low- and middle-income countries
MEDLINE: Medical Literature Analysis and Retrieval System Online
PRISMA: Preferred Reporting Items for Systematic Reviews and Meta-analyses
PsychINFO: Psychological Information Database
